# Sleep Disturbances Among Pregnant Women Attending a Maternity Teaching Hospital in Erbil, Iraq

**DOI:** 10.7759/cureus.71088

**Published:** 2024-10-08

**Authors:** Srwa Abdulrahman Mustafa, Sahar Ismail Abdulla, Tiran Jamil Piro, Wahida Abdulla Ibrahim, Shaymaa Sameer Maqsood, Abdulmalik F Saber

**Affiliations:** 1 Department of Nursing, College of Nursing, Hawler Medical University, Erbil, IRQ; 2 Department of Nursing, Faculty of Nursing, Tishk International University, Erbil, IRQ

**Keywords:** maternal health, pregnancy, pregnant women, sleep disturbance, sleep quality

## Abstract

Background and aim

Sleep disturbances during pregnancy are a common occurrence and can significantly impact both maternal and fetal health. Therefore, this study aimed to assess the severity of sleep disturbances among pregnant women attending the Erbil Maternity Teaching Hospital in Erbil, Iraq.

Method

This cross-sectional study was conducted from July 15, 2023, to August 15, 2024, at the Erbil Maternity Teaching Hospital in Erbil using convenience sampling. Data were gathered using a self-structured questionnaire that included demographic information and a 20-item sleep disturbance assessment. Statistical analysis was performed using Stata version 12 (StataCorp LLC, College Station, TX), and parametric tests such as the t-test, ANOVA, and multiple linear regression were used to examine the relationships between sleep disturbances and demographic variables. A p-value of less than 0.001 was considered statistically significant.

Results

A total of 300 participants were enrolled in the study. The mean sleep disturbance score was 47.58 ± 9.83, indicating that most participants experienced mild sleep disturbances. Sleep disturbances did not significantly differ by age, education level, occupation, or residency. However, economic status was significantly associated with sleep disturbance levels, with lower-income participants experiencing more severe disturbances (p = 0.02).

Conclusions

The study found that most pregnant women experienced mild sleep disturbances, with lower economic status being a significant predictor of more severe sleep issues. Healthcare providers and policymakers are recommended to develop targeted interventions that address the unique needs of pregnant women, particularly those from lower-income groups, to improve sleep quality and overall maternal health outcomes.

## Introduction

Sleep disturbances during pregnancy are a common occurrence, affecting a significant proportion of expectant mothers worldwide [1]. These disturbances can manifest in various forms, such as difficulty falling asleep, frequent nighttime awakenings, and poor sleep quality [2]. Pregnancy is a unique period characterized by numerous physical, hormonal, and emotional changes that can significantly impact sleep patterns and overall well-being [3]. The prevalence of sleep disturbances during pregnancy has been reported to range from 46% to 78% across different studies and populations [4]. The factors contributing to sleep disturbances in pregnant women are multifaceted and can vary depending on the stage of pregnancy [5,6]. During the first trimester, hormonal changes, particularly an increase in progesterone levels, can lead to daytime sleepiness and increased sleep fragmentation [7]. As the pregnancy progresses, physical discomforts such as back pain, leg cramps, and frequent urination become more prevalent, further disrupting sleep [8]. In addition to these physical changes, psychological factors, including stress, anxiety, and depression, can contribute to sleep disturbances throughout pregnancy [7,9-11].

The consequences of sleep disturbances during pregnancy extend beyond mere discomfort and can have significant implications for both maternal and fetal health [7,11]. For instance, inadequate sleep has been associated with an increased risk of gestational diabetes, preeclampsia, and prolonged labor [12]. Moreover, sleep disturbances can negatively impact mood, cognitive function, and overall quality of life during pregnancy [13]. Poor sleep quality has also been linked to a higher likelihood of postpartum depression, which can have long-term effects on maternal well-being and mother-infant bonding [14]. Despite the high prevalence and potential consequences of sleep disturbances during pregnancy, this issue often remains underrecognized and undertreated. This lack of attention may be due to the fact that many pregnant women consider sleep disturbances as an inevitable part of pregnancy and may not seek help or discuss their concerns with healthcare providers [15]. Additionally, healthcare professionals may not routinely screen for sleep problems or provide adequate education and support to expectant mothers regarding sleep hygiene and management strategies [16].

Given the potential impact of sleep disturbances on maternal and fetal health outcomes, it is crucial to investigate the prevalence and characteristics of sleep problems among pregnant women in different settings and populations. While several studies have examined sleep disturbances during pregnancy in various countries, there is limited research focusing on pregnant women in Iraq, particularly in the city of Erbil. Thus, understanding the specific sleep patterns, contributing factors, and associated consequences among pregnant women in this region is essential for developing targeted interventions and improving maternal and fetal health outcomes. In particular, addressing psychological and behavioral approaches to improve sleep in pregnant women is critical. Cognitive behavioral therapy (CBT) is a well-established approach effective in managing various psychological conditions, including anxiety, depression, and even suicidal ideation [17]. Recently, CBT has also been applied to sleep disorders, particularly insomnia [18]. Among pregnant women, CBT addresses both cognitive and behavioral factors contributing to sleep disturbances, helping manage the anxiety and stress associated with pregnancy [18]. By teaching relaxation techniques and sleep hygiene practices, CBT can significantly improve sleep quality, making it a valuable intervention for pregnant women experiencing sleep issues.

Moreover, exploring sleep disturbances among pregnant women attending a maternity teaching hospital in Erbil, Iraq, can provide valuable insights into the unique challenges and needs of this population. Teaching hospitals often serve a diverse patient population and play a crucial role in providing comprehensive maternal care and education [19-21]. By addressing the gaps in sleep care within these institutions, researchers can identify potential deficiencies in screening, diagnosis, and management of sleep problems during pregnancy. This information can inform the development of evidence-based guidelines and training programs for healthcare professionals, ultimately improving the quality of care provided to pregnant women. Therefore, this study aimed to assess the severity of sleep disturbances among pregnant women attending the Erbil Maternity Teaching Hospital in Erbil, Iraq.

## Materials and methods

Study design, setting, period, and sampling

This cross-sectional study was conducted in Erbil, Iraq, at the Erbil Maternity Teaching Hospital. The convenience sampling method was used to collect data from July 15, 2023, until August 15, 2024.

Sample size

To calculate the required sample size for this correlation study, we used the parameters of a 5% margin of error, a 95% confidence interval, and a population proportion of 50%. Given that the total number of pregnant women attending the Erbil Maternity Teaching Hospital in Erbil City was approximately 1,350, the population size was set at 1,350. After considering these factors, the final sample size was determined to be 300 cases.

Inclusion/exclusion criteria

The study's inclusion criteria were pregnant women of any age who were admitted to the Erbil Maternity Teaching Hospital in Erbil City and were willing to participate in the study. Exclusion criteria included women with documented psychological disorders, those with chronic medical conditions that significantly impact sleep (e.g., severe hypertension and diabetes), and those in their first trimester of pregnancy, as sleep disturbances are less prevalent in early pregnancy.

Study tools and data collection

The questionnaire was divided into two main parts. The first part gathered demographic data, including age group (years), education level, occupation, residency, and economic status. The second part was a self-structured questionnaire containing 20 items designed to assess the levels of sleep disturbance. The questionnaire was originally developed in English and then translated into Kurdish using the forward-backward method. The translation was verified by 10 nursing professors from different specialties. Data were collected by distributing questionnaires to participants who met the inclusion criteria. Each participant was given 10-15 minutes to complete the questionnaire.

Pilot study

The study questionnaire was initially tested with a group of 30 participants from January 2nd to February 3rd, 2023, to assess the internal consistency and reliability of its items before their use in the actual study. The internal consistency was calculated using Cronbach's alpha [22], yielding a value of 0.81, which indicates a very good level of internal consistency and reliability. For content validity, the questionnaire was reviewed by 10 nursing professors in the field to ensure its appropriateness. It is important to note that the data from this initial study were excluded from the final analysis.

Measures

Sociodemographic Characteristics

The first section of the questionnaire included sociodemographic information about pregnant women, such as age group (years), education level, occupation, residency, and economic status.

Sleep Disturbance Self-Structured Questionnaire

To assess sleep disturbances among pregnant women, we used a self-structured questionnaire consisting of 20 items. The responses were divided into five categories: never, rarely, occasionally, mostly, and always. The scoring was designed to assess the severity of sleep disturbances, with the total score categorized into four levels: normal (1-25), mild (26-50), moderate (51-75), and severe (76-100). This categorization was determined by summing up the responses to all questions, with higher scores indicating greater severity of sleep disturbances. The reliability of the questionnaire was thoroughly evaluated using Cronbach's alpha [16], which resulted in a very good alpha coefficient of 0.81, indicating high reliability.

Ethical approval and informed consent

This study followed the Institutional Research Ethics Board and the Declaration of Helsinki guidelines. Ethical approval for this study was obtained on June 20, 2023 (approval number: 8) from the Ethics Committee of Hawler Medical University. Oral informed consent was obtained from all participants before they filled out the questionnaires.

Statistical analysis

Data were summarized and reported with frequency and percentage for qualitative variables. Quantitative variables were presented with mean and standard deviations. The data were weighted to the population and standardized according to the WHO population estimates for 2000-2025 using survey analysis. Given that our data are normally distributed, we adhered to parametric tests such as the t-test and ANOVA, followed by multiple linear regression. The t-test and ANOVA were used to assess the relationship between sleep disturbances and confounding variables. To further ensure the relationship, multiple linear regression analysis was performed. Data analysis was conducted using Stata version 12 (StataCorp LLC, College Station, TX), with significance levels considered at P < 0.001.

## Results

Demographic characteristics and sleep disturbance levels

A total of 300 pregnant women participated in the study. The participants' ages ranged from 16 to 45 years, with a mean age of 27.50 ± 6.40 years. Of the total, the majority of the participants, 135 (45.0%), were aged between 16 and 25 years, while those aged 26-35 years comprised 122 (40.7%) of the sample. In addition to age distribution, educational attainment varied, with 68 (22.7%) participants having a secondary school education, followed by 66 (22.0%) with a primary school education. Moreover, most participants, 272 (90.7%), were housewives, with only 28 (9.3%) being employed. Regarding residency, the majority, 228 (76.0%), lived in urban areas, while 72 (24.0%) resided in rural areas. Economic status further illustrated the sample diversity, as 222 (74.0%) reported having a medium income, 45 (15.0%) had a low income, and 33 (11.0%) reported a high income. With respect to sleep patterns, 182 (60.6%) participants experienced mild sleep disturbances, 114 (38.0%) had moderate sleep disturbances, and only two (0.7%) reported severe or normal sleep patterns. The overall mean sleep disturbance score was 47.58 ± 9.83, indicating that most participants experienced mild sleep disturbances. For further demographic and sleep-related insights, please refer to Table 1.

**Table 1:** Demographic characteristics, socioeconomic status, and sleep disturbance levels among pregnant women attending the Maternity Teaching Hospital in Erbil City (n = 300). Note: Results are expressed as N (%) and mean ± SD. N = number; % = percentage; SD = standard deviation.

Items	Categories (n = 300)	N	%
Age (year)	16-25	135	45.0
	26-35	122	40.7
	36-45	43	14.3
	Mean ± SD	27.50 ± 6.40	
Education level	Illiterate	43	14.3
	Primary school	66	22.0
	Secondary school	68	22.7
	High school	49	16.3
	Vocational/institution student	16	5.3
	College student	10	3.3
	Graduate	48	16.0
Occupation	Employee	28	9.33
	Housewife	272	90.67
Residency	Urban	228	76.0
	Rural	72	24.0
Economical state	Low	45	15.0
	Medium	222	74.0
	High	33	11.0
Sleep disturbance levels	Normal	2	0.7
	Mild	182	60.6
	Moderate	114	38.0
	Severe	2	0.7
	Mean ± SD	47.58 ± 9.83	

Sleep disturbance items

The participants reported varying levels of sleep disturbances across multiple factors. When asked about difficulty falling asleep, 117 (39.0%) always experienced this issue, while 60 (20.0%) reported it mostly, indicating a common problem. In contrast, 294 (98.0%) never took any medication or substances to aid sleep, and 300 (100%) reported they never used alcohol for sleep. Regarding medical conditions affecting sleep, 247 (82.3%) participants reported no disruption, while 14 (4.7%) experienced this always. Emotional factors also played a role in sleep disturbances, with 82 (27.3%) always losing interest in previous hobbies or activities, and 73 (24.3%) always feeling sad, irritable, or hopeless. Nervousness or anxiety was cited by 75 (25.0%) as always impacting their sleep, while 181 (60.3%) were never concerned that something might be physically wrong with their baby disrupting sleep. In terms of physical discomfort, 123 (41.0%) always experienced restlessness or discomfort in the legs before sleep. Additionally, 213 (71.0%) had never been told they moved excessively or kicked during sleep, though 73 (24.3%) reported always engaging in unusual behaviors like sleepwalking or talking. Snoring was not prevalent, with 245 (81.7%) reporting no snoring, but 32 (10.7%) always exhibited symptoms such as stopping breathing or gasping during sleep. Daytime consequences of poor sleep were evident, as 93 (31.0%) always felt drowsy during the day, and 43 (14.3%) found that work or other activities consistently prevented sufficient sleep. Nightmares and medication-related sleep disturbances were relatively rare, with 139 (46.3%) never experiencing nightmares and 291 (97.0%) reporting no issues with medication disturbing their sleep. Finally, nocturia (frequent urination) was a significant disturbance, with 163 (54.3%) always being woken up during the night due to the need to urinate. For further details, refer to Table 2.

**Table 2:** Frequency and percentage distribution of sleep disturbance variables among pregnant women attending the Maternity Teaching Hospital (N = 300). Note: N = 300 is the total number of participants in the study; results are expressed as N (%). N = number; % = percentage.

Items	Never		Rarely		Occasionally		Mostly		Always	
	N	%	N	%	N	%	N	%	N	%
Do you have difficulty falling asleep?	53	17.7	15	5.0	55	18.3	60	20.0	117	39.0
Do you take any medication or substances to help you sleep?	294	98.0	0	0.0	1	0.3	0	0.0	5	1.7
Do you consume alcohol to aid your sleep?	300	100.0	0	0.0	0	0.0	0	0.0	4	1.3
Do you have any medical conditions that disrupt your sleep?	247	82.3	10	3.3	18	6.0	11	3.7	14	4.7
Have you lost interest in activities or hobbies that you previously enjoyed?	111	37.0	24	8.0	40	13.3	43	14.3	82	27.3
Do you often feel sad, irritable, or hopeless, affecting your sleep?	56	18.7	40	13.3	83	27.7	48	16.0	73	24.3
Do you feel nervous or anxious, which impacts your sleep?	71	23.7	36	12.0	64	21.3	54	18.0	75	25.0
Do you think there is something physically wrong with your body that affects your sleep?	146	48.7	33	11.0	61	20.3	33	11.0	27	9.0
Are you concerned that something may be wrong with your baby, disrupting your sleep?	181	60.3	21	7.0	42	14.0	20	6.7	36	12.0
Is your sleep schedule irregular due to work or other activities?	135	45.0	8	2.7	40	13.3	42	14.0	75	25.0
Do you experience restlessness or discomfort in your legs before going to bed?	59	19.7	13	4.3	59	19.7	46	15.3	123	41.0
Have others told you that you move a lot or kick your legs during sleep?	213	71.0	18	6.0	23	7.7	19	6.3	27	9.0
Do you exhibit unusual behaviors or movements during sleep (e.g., sleepwalking and talking)?	125	41.7	41	13.7	41	13.7	20	6.7	73	24.3
Do you snore while sleeping?	245	81.7	17	5.7	22	7.3	9	3.0	7	2.3
Has anyone mentioned that you stop breathing, gasp, snort, or choke during your sleep?	169	56.3	20	6.7	43	14.3	36	12.0	32	10.7
Do you feel drowsy or sleepy during the day?	38	12.7	34	11.3	63	21.0	72	24.0	93	31.0
Do work or other daytime activities prevent you from getting enough sleep?	187	62.3	14	4.7	42	14.0	14	4.7	43	14.3
Do you experience frequent nightmares that disturb your sleep?	139	46.3	43	14.3	48	16.0	45	15.0	25	8.3
Has the medication you are taking disturbed your sleep?	291	97.0	1	0.3	2	0.7	1	0.3	5	1.7
Does frequent urination (nocturia) wake you up during the night?	31	10.3	21	7.0	38	12.7	47	15.7	163	54.3

Relationship between sleep disturbance with demographic data

The participants' sleep disturbance scores did not significantly differ by age (p = 0.33), with mean scores ranging from 47.16 to 49.52 across age groups. Similarly, education level showed no significant differences in sleep disturbance scores (p = 0.41), though college students had the highest mean score (55.70). Occupation (p = 0.70) and residency (p = 0.72) also showed no significant impact on sleep disturbance, with housewives and urban residents having slightly higher scores. However, economic status was significantly associated with sleep disturbance (p = 0.02), with participants in the low-income group reporting the highest mean score (50.56) (Table 3).

**Table 3:** Mean scores and standard deviations of sleep disturbance variables by demographic factors among pregnant women (n = 300). Note: N = 300 is the total number of participants in the study; results are expressed as N (%) and mean score (MS). ^a^ Independent sample t-test (T) and ^b^ one-way ANOVA test (F) were used to assess the relationship between sleep disturbance levels and demographic data. Significance was set at P < 0.001.

			Sleep disturbance			
Variables	Items	Number	MS	SD	T\F	P-value
Age (year)	16-25	135	47.25	9.70	1.18^b^	0.33
	26-35	122	47.16	9.76		
	36-45	43	49.52	10.31		
Education level	Illiterate	43	48.42	11.09	2.65^b^	0.41
	Primary school	66	46.62	9.58		
	Secondary school	68	48.93	9.75		
	High school	49	46.39	8.93		
	Vocational/institution student	16	50.69	11.72		
	College student	10	55.70	9.38		
	Graduate	48	44.73	8.25		
Occupation	Employee	28	46.89	9.57	-0.39^a^	0.70
	Housewife	272	47.65	9.87		
Residency	Urban	228	47.69	9.75	0.35^a^	0.72
	Rural	72	47.22	10.12		
Economical state	Low	45	50.56	10.33	4.13^b^	0.02
	Medium	222	47.48	9.69		
	High	33	44.19	9.07		

Regression correlations with demographic data

The analysis showed that age was not a significant predictor of sleep disturbance (p = 0.17), with a standardized coefficient (B) of 0.08 and a 95% confidence interval ranging from -0.49 to 2.72. Similarly, education level (p = 0.10), occupation (p = 0.75), and residency (p = 0.69) were not significantly associated with sleep disturbance. However, economic status was a significant predictor (p = 0.04), with a standardized coefficient (B) of -0.17, indicating that participants with lower economic status had higher sleep disturbance scores. The 95% confidence interval for economic status ranged from -5.48 to -1.02. For further details, refer to Table 4.

**Table 4:** Final model of multiple linear regression for assessing the association between sleep disturbance and demographic variables. Note: Sleep disturbance is the dependent variable. Significance was set at P < 0.001.

Variables	Coefficient standardized (B)	Coefficient unstandardized (B)	95% confidence interval		P-value
			Lower	Upper	
Age	0.08	1.11	-0.49	2.72	0.17
Education level	0.00	0.00	-0.65	0.66	0.10
Occupation	0.02	0.72	-3.64	5.08	0.75
Residency	-0.02	-0.54	-3.15	2.07	0.69
Economical state	-0.17	-3.25	-5.48	-1.02	0.04

## Discussion

This study aimed to assess the severity of sleep disturbances among pregnant women attending the Erbil Maternity Teaching Hospital in Erbil, Iraq. The results not only revealed the prevalence of sleep disturbances but also highlighted varying degrees of severity among participants. Overall, the results indicated that the majority of participants experienced mild sleep disturbances, with a smaller proportion reporting moderate to severe disturbances.

Sleep disturbances during pregnancy are a common issue that can have significant implications for both maternal and fetal health, potentially leading to complications such as gestational diabetes, hypertension, and preterm birth [23]. Given the potential health risks, addressing sleep issues in pregnancy is critical, especially in regions with unique challenges like Iraq. Despite the well-documented prevalence of sleep problems in pregnancy, there is limited research specifically addressing these issues among pregnant women in Iraq. The unique cultural norms, socioeconomic challenges, and healthcare disparities in this region underscore the importance of investigating sleep disturbances and their associated factors in this context. Additionally, factors like economic status and living conditions may uniquely influence sleep patterns in this population, necessitating a more targeted approach. Understanding how local factors like economic status and living conditions impact sleep patterns is crucial for developing targeted interventions. Given the critical nature of these aspects, our study aimed to provide comprehensive insights into the severity and predictors of sleep disturbances among pregnant women in Erbil.

The demographic characteristics of our study participants, primarily young housewives living in urban areas with varying economic statuses, provide a representative sample of the pregnant population attending the Erbil Maternity Teaching Hospital. This demographic snapshot is essential for contextualizing the findings of our study within the broader landscape of pregnant women in Erbil. The majority of participants being in the 16-25 years age range aligns with the typical childbearing age in Iraq [24]. The high proportion of housewives in our sample reflects the cultural norms and gender roles in the region, where women often prioritize family responsibilities over employment [24]. The economic status of our participants, with a large portion reporting medium incomes and smaller groups identifying as low- or high-income earners, reflects the socioeconomic diversity of the region. This distribution is comparable to the general economic landscape in Iraq [24,25]. Interestingly, economic status emerged as a significant factor, as our study found that participants with lower economic status experienced higher levels of sleep disturbance. This suggests that financial stress may contribute to poor sleep quality, consistent with previous research identifying socioeconomic status as a significant predictor of sleep disturbances during pregnancy [26].

The prevalence of mild sleep disturbances among our participants is consistent with findings from studies conducted in other countries [27,28]. However, the slightly higher proportion of moderate to severe disturbances in our sample compared to global averages warrants further exploration. This difference may be attributed to the unique stressors and challenges faced by pregnant women in the context of Iraq, such as political instability, limited healthcare resources, and cultural expectations. The specific sleep disturbances reported by our participants, such as difficulty falling asleep, restlessness or discomfort in the legs, and frequent urination at night, are consistent with the common sleep complaints during pregnancy [29]. Notably, the low prevalence of medication or substance use to aid sleep is a positive outcome, as the use of these substances during pregnancy can have adverse effects on fetal development [30]. The infrequent use of alcohol among our participants is also encouraging, as alcohol consumption during pregnancy is associated with a range of risks.

The role of emotional factors, such as sadness, irritability, and anxiety, in contributing to sleep disturbances for a notable number of participants highlights the importance of addressing mental health during pregnancy. This underscores the need for integrating mental health support into prenatal care, particularly for managing sleep issues. This finding aligns with previous research that has established the bidirectional relationship between sleep disturbances and perinatal mood disorders [31]. The relatively low prevalence of concerns about physical issues impacting the baby is a positive finding, as excessive worry and anxiety can exacerbate sleep disturbances [32]. Moreover, our study did not find significant differences in sleep disturbance scores based on factors like age, education, or occupation, which contrasts with some prior research. The lack of significant differences in sleep disturbance scores based on age, education level, occupation, and residency suggests that these factors may not be primary determinants of sleep quality among pregnant women in Erbil. This finding contrasts with some previous studies that have identified associations between these variables and sleep disturbances [33,34]. The discrepancy may be attributed to the specific cultural and socioeconomic context of our study setting, where other factors, such as economic status, may play a more prominent role in influencing sleep patterns.

While this study provides valuable insights into sleep disturbances among pregnant women in Erbil, several limitations should be acknowledged. Addressing these limitations in future research could further enhance the understanding of sleep issues during pregnancy in this population. The cross-sectional design limits the ability to establish causal relationships between the investigated variables and sleep disturbances. Additionally, the reliance on self-reported data may introduce recall bias or social desirability bias. Future research should consider employing longitudinal designs and objective sleep measures, such as actigraphy, to further explore the dynamics of sleep disturbances throughout pregnancy. Moreover, investigating the impact of sleep disturbances on maternal and fetal outcomes in this population would provide a more comprehensive understanding of the implications of this issue.

## Conclusions

The study revealed that most pregnant women experience mild sleep disturbances, with lower economic status being a significant predictor of more severe sleep issues. It is recommended that healthcare providers and policymakers create targeted interventions that address the specific needs of pregnant women, especially those from lower-income groups, to enhance sleep quality and overall maternal health outcomes. These initiatives should include routine screening for sleep disturbances and customized support for managing sleep issues during pregnancy.

## Appendices

Questionnaire

This questionnaire is integral to our article titled "Sleep Disturbances Among Pregnant Women Attending Maternity Teaching Hospital in Erbil, Iraq." Your participation is entirely voluntary and highly valued. The questionnaire guarantees complete anonymity; hence, please do not provide any personal identifiers such as your name. Your insights are important to this study, and I am grateful for the time you dedicate to completing this survey.

Code …………

**Table 5:** Demographic characteristics. Respondents are asked to tick the option that fits them for each category.

Part 1: Sociodemographic characteristics	Options	Response
X1: Age	16-25	
	26-35	
	36-45	
X2: Educational level	Illiterate	
	Primary school	
	Secondary school	
	High school	
	Vocational/institution student	
	College student	
	Graduate	
X3: Occupation	Employee	
	Housewife	
X4: Residency	Urban	
	Rural	
X5: Economical state	Low	
	Medium	
	High	

Part 2: Sleep disturbance questionnaire

Please read each statement and circle a number from 1 to 5, which indicates how much the statement applies to you. There are no right or wrong answers. Do not spend too much time on any statement.

**Table 6:** Sleep disturbance questionnaire. Respondents are asked to rate each statement based on how much it applies to them. The rating scale is as follows: 1 - never, 2 - rarely, 3 - occasionally, 4 - mostly, and 5 - always.

Item No.	Statement	Never	Rarely	Occasionally	Mostly	Always
1.	Do you have difficulty falling asleep?	1	2	3	4	5
2.	Do you take any medication or substances to help you sleep?	1	2	3	4	5
3.	Do you consume alcohol to aid your sleep?	1	2	3	4	5
4.	Do you have any medical conditions that disrupt your sleep?	1	2	3	4	5
5.	Have you lost interest in activities or hobbies that you previously enjoyed?	1	2	3	4	5
6.	Do you often feel sad, irritable, or hopeless, affecting your sleep?	1	2	3	4	5
7.	Do you feel nervous or anxious, which impacts your sleep?	1	2	3	4	5
8.	Do you think there is something physically wrong with your body that affects your sleep?	1	2	3	4	5
9.	Are you concerned that something may be wrong with your baby, disrupting your sleep?	1	2	3	4	5
10.	Is your sleep schedule irregular due to work or other activities?	1	2	3	4	5
11.	Do you experience restlessness or discomfort in your legs before going to bed?	1	2	3	4	5
12.	Have others told you that you move a lot or kick your legs during sleep?	1	2	3	4	5
13.	Do you exhibit unusual behaviors or movements during sleep (e.g., sleepwalking and talking)?	1	2	3	4	5
14.	Do you snore while sleeping?	1	2	3	4	5
15.	Has anyone mentioned that you stop breathing, gasp, snort, or choke during your sleep?	1	2	3	4	5
16.	Do you feel drowsy or sleepy during the day?	1	2	3	4	5
17.	Do work or other daytime activities prevent you from getting enough sleep?	1	2	3	4	5
18.	Do you experience frequent nightmares that disturb your sleep?	1	2	3	4	5
19.	Has the medication you are taking disturbed your sleep?	1	2	3	4	5
20.	Does frequent urination (nocturia) wake you up during the night?	1	2	3	4	5
